# Anti-*Helicobacter pylori* Activity of Isocoumarin Paepalantine: Morphological and Molecular Docking Analysis

**DOI:** 10.3390/molecules22050786

**Published:** 2017-05-12

**Authors:** João Paulo L. Damasceno, Ricardo P. Rodrigues, Rita de Cássia R. Gonçalves, Rodrigo R. Kitagawa

**Affiliations:** 1 Graduate Program in Pharmaceutical Sciences, Federal University of Espirito Santo–UFES, Marechal Campos Av., 1468, Vitoria 29043-900, ES, Brazil; jpldamasceno@yahoo.com.br (J.P.L.D.); ricardo.p.rodrigues@ufes.br (R.P.R.); rita.goncalves@ufes.br (R.d.C.R.G.); 2 Department of Pharmaceutical Sciences, Federal University of Espirito Santo–UFES, Marechal Campos Av., 1468, Vitoria 29043-900, ES, Brazil

**Keywords:** paepalantine, isocoumarin, antibacterial, *Helicobacter pylori*, penicillin-binding protein, docking

## Abstract

The *Helicobacter* *pylori* bacterium is one of the main causes of chronic gastritis, peptic ulcers, and even gastric cancer. It affects an average of half of the world population. Its difficult eradication depends upon multi-drug therapy. Since its classification as a group 1 carcinogenic by International Agency for Research on Cancer (IARC), the importance of *H. pylori* eradication has obtained a novel meaning. There is considerable interest in alternative therapies for the eradication of *H. pylori* using compounds from a wide range of natural products. In the present study, we investigated the antibacterial property of the isocoumarin paepalantine against *H. pylori* and it exhibited significant anti-*H. pylori* activity at a minimum inhibitory concentration (MIC) of 128 μg/mL and at a minimum bactericidal concentration (MBC) of 256 μg/mL. The scanning electron microscopy (SEM) revealed significant morphological changes of the bacterial cell as a response to a sub-MIC of paepalantine, suggesting a penicillin-binding protein (PBP) inhibition. Computational studies were carried out in order to study binding modes for paepalantine in PBP binding sites, exploring the active and allosteric sites. The data from the present study indicates that paepalantine exhibits significant anti-*H. pylori* activity, most likely by inhibiting membrane protein synthesis.

## 1. Introduction

*Helicobacter pylori* is a gram-negative bacterium that infects the gastric mucosa and is considered one of the main etiological agents of chronic gastritis, eventually leading to the development of peptic ulcers and gastric cancer [1,2,3] .The mechanisms by which *H. pylori* may cause gastroduodenal disease and contributes to gastric carcinogenesis are not fully elucidated. However, the production of specific virulence factors by the bacterium, the host’s inflammatory response, and the association with environmental contributors may all be responsible [4].

The mechanism of action of the bacterium comprises the establishment of *H. pylori* in the stomach, inducing inflammatory responses, and pathological changes in the gastric microenvironment. Neutrophils and monocytes are recruited to the site of infection, where they produce reactive oxygen species (ROS) and nitrogen species (RNS) [2,5]. However, *H. pylori* disrupts NADPH oxidase targeting, consequently, superoxide anions are released into the extracellular medium rather than accumulated within bacteria-containing phagosomes, thus contributing to the induction of host tissue damage and ulceration [6]. The *H. pylori’s* ability to avoid the immune response leads to a local persistent inflammation, which, in turn, results in large amounts of ROS and RNS [7].

The prevalence of *H. pylori* infection is still high in most countries. It ranges from 40% in developed countries up to 80% in underdeveloped countries. According to estimates, 10 to 20% of *H. pylori*-infected patients develop different degrees of peptic ulcer diseases, and approximately 1–2% are at risk of developing stomach cancer [2,8].

The standard treatment for *H. pylori* infection eradication combines three drugs. Two are antimicrobial agents, including amoxicillin, clarithromycin and/or metronidazole, and the third drug is bismuth or a proton pump inhibitor [9,10]. Nevertheless, triple therapy is not always successful because of the emergence of resistant *H. pylori* strains and side effects of the current chemotherapeutic approach [11,12]. For these reason, several studies have been undertaken to develop new drugs that might act as an alternative treatment for *H. pylori* infection [13,14,15,16].

In this context, natural products have always played a significant role in the discovery of drugs, including antibiotics. More than one-third of all the molecules approved by the Food and Drug Administration (FDA) are of natural origin, and their derivatives are semisynthetic [4,16,17]. Among the numerous natural product classes, coumarins have emerged as an important, widely distributed phytochemical class with diverse pharmacological effects, such as antitumor [18], anticoagulant [19], anti-inflammatory [20], anti-oxidant [21], and antimicrobial actions [22] as antibiotics novobiocin and its coumarins derivatives [23,24,25,26].

The isocoumarin paepalantine (9,10-dihydroxy-5,7-dimethoxy-3-methyl-1*H*-naphtho[2,3-*c*]pyran-1-one), isolated from the capitula of Paepalanthus bromelioides (SILV.) [27], has proven to have a wide variety of biological activities, amongst which are antibacterial [28,29], anti-oxidant [30,31], anti-inflammatory [32], and cytotoxic actions [33].

Thus, based on the previous studies with paepalantine which showed antibacterial and antioxidant potential, the objective of the present study was to investigate the effect of paepalantine on the viability and morphology of *H. pylori*, conducting in vitro and in silico analysis of possible molecular targets.

## 2. Results

### 2.1. Anti-H. pylori Activity

**Figure 1:**
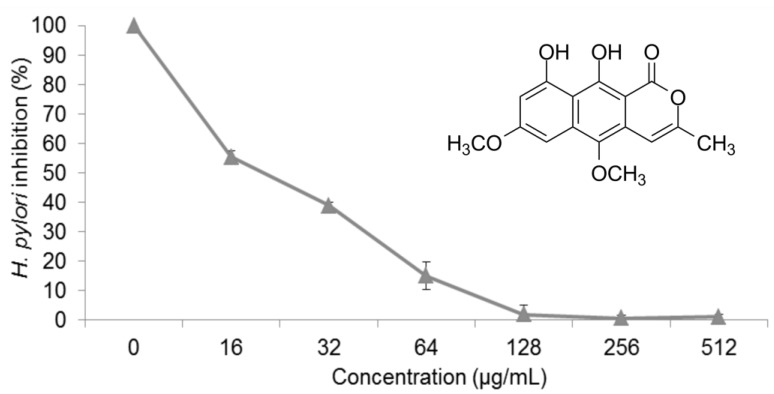
Effect of paepalantine on *H. pylori* growth after 72 h of incubation.

Figure 1 shows the effect of paepalantine on *H. pylori*; paepalantine showed a MIC of 128 μg/mL and a minimum bactericidal concentration (MBC) of 256 μg/mL. The vehicle used (propylene glycol) had no inhibitory effect on the micro-organisms at the concentrations used in the assay. Since the bacterial strain used in this assay was amoxicillin sensitive and metronidazole resistant, only amoxicillin presented MIC and MBC values, which were 0.25 μg/mL and 2 μg/mL, respectively.

**Figure 2:**
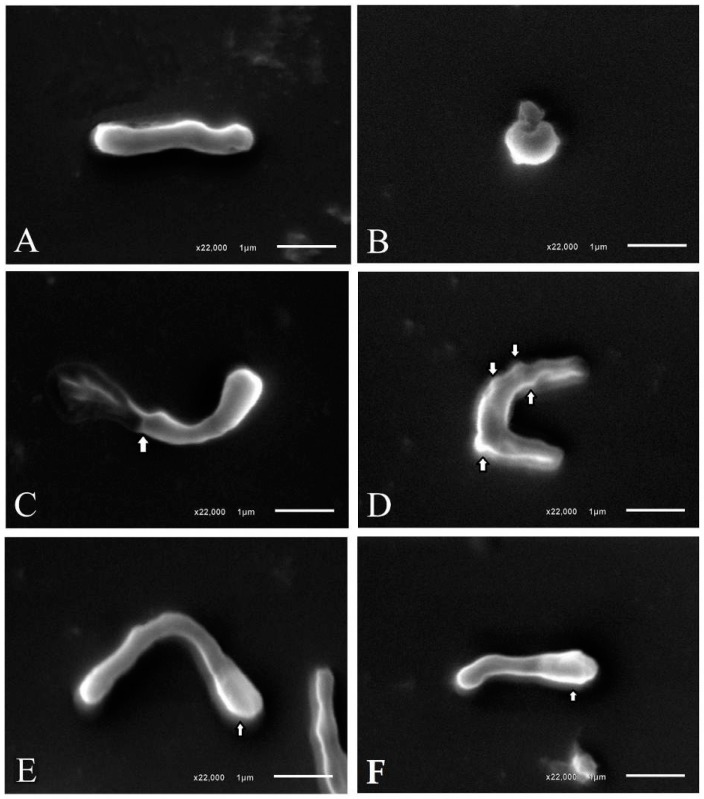
Scanning electron micrographs of *H. pylori* treated with paepalantine MIC and sub-MIC. (**A**) *H. pylori* control bacteria; (**B**) Spherical cell formation seen after treatment with paepalantine MIC; (**C**) Damaged bacteria after treatment with paepalantine MIC; (**D**) Membrane blebbing (indicated by the arrowhead) seen after treatment with paepalantine sub-MIC; (**E**,**F**) Tendency to spherical polarization (indicated by the arrowhead) seen after treatment with paepalantine sub-MIC.

The effect of paepalantine on *H. pylori* growth and viability led us to study bacterial morphology after exposure to a MIC and sub-MIC. The treatment of *H. pylori* with CIM and ½ MIC (64 μg/mL) of paepalantine resulted in morphological changes in the membrane that were not observed in the control. In the control treatment, the *H. pylori* cells presented regular shapes with even surfaces, whereas the cells treated with paepalantine were damaged and exhibited altered or irregular shapes, such as spheres and cell surface blebs (Figure 2). These morphological changes were also observed in previous studies [34,35] in *H. pylori* treated with β-lactam antibiotics, which correlated the performance of these antibiotics to penicillin-binding proteins (PBPs).

### 2.2. Computational Methods

**Figure 3:**
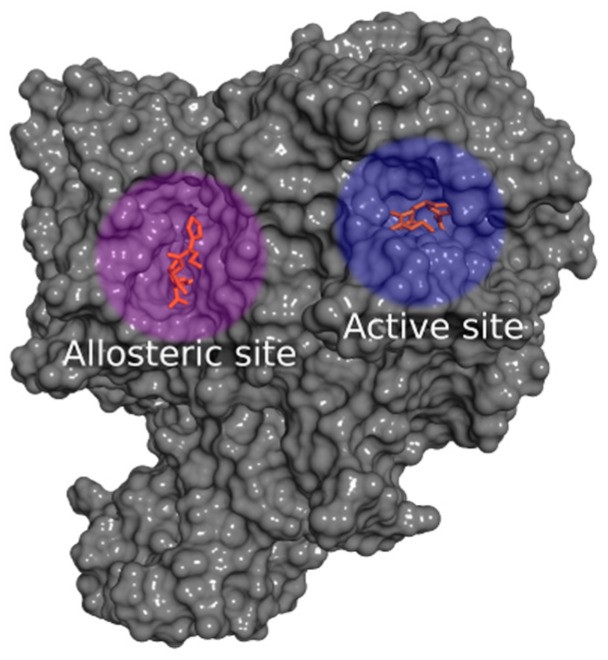
Surface representation of the crystal structure of the protein-ligand complex of the Penicillin-binding protein (PDB code: 1QMF), evidencing the cavities of the active site and the allosteric site.

The development of the docking model was based on the analysis of the crystallographic chemical structures of the available penicillin-binding proteins complexed with betalactamic inhibitors, obtained from the Protein Data Bank (PDB) database [36,37] (Table 1, Figure 3). The PDB code, 1QMF, was selected for docking analysis, since it contains preserved information of the cefuroxime acylation mechanism in its active site and a non-covalent binding of cefuroxime in the allosteric site, allowing for study and comprehension of both binding interactions. The other PDB codes did not allow for these conditions since the allosteric binding site was not preserved.

Penicillin-binding proteins (PBPs) have an important hole in the maintenance of the bacterial cell wall. The penicillin-binding proteins with an allosteric site binding mechanism promotes a conformational change during its binding that results in a slight opening of the active site, making it more accessible for substrate binding. Finding inhibitors with affinity for both the active and allosteric site is a promising strategy for drug discovery. In order to properly evaluate the affinity of paepalantine for PBPs, it was necessary to investigate both the active and allosteric binding mechanism, since this investigation could result in an additional and unique inhibition pattern [15,38].

#### 2.2.1. Docking Calculations

Molecular docking calculations were performed using the Molecular Operating Environment suite (MOE) [39]. For docking calculations, the placement method was set to the pharmacophore search engine. The scoring function was set to “London dG”, which estimates the free energy of binding of the ligand from a given pose. A set of 30 docking poses for each molecule were retained during the initial calculation and then refined by energy minimization using the molecular mechanics force field method and then submitted to a second refinement, using the “GBVI/WSA dG” scoring function, selecting the top 10 docking poses for each ligand [40].

#### 2.2.2. Docking Validation and Development of the Model

The pose prediction accuracy of the docking software was evaluated by redocking analysis. The redocking process consists of a high-quality structural model of the protein structure, usually extracted from an X-ray crystal of a protein-ligand complex. Then, the co-crystal ligand is extracted from this file, leaving only the protein that is prepared for docking calculation. Once the calculation is performed, the docking poses were compared to the protein-ligand complex. The measure of this deviation is represented by the root mean square deviation (RMSD) measure. Values below 2 Å deviation are considerable acceptable [41,42]. The protein-ligand complex was obtained from the PDB database (PDB code 1QMF). The RMSD values from the redocking process for the binding site and allosteric site were respectively, 1.14 Å and 1.31 Å, and were considered satisfactory for pose prediction (For more information, access the available supplementary material).

The developed model was based on the inhibitor cefuroxime, since it is the smallest co-crystallized ligand available amongst the other two ligands, tebipenem and biapenem (Table 1). As a result of its small volume, cefuroxime is also able to explore both the active and the allosteric site. Cefuroxime is also similar in size/shape with the investigated metabolite paepalantine. After the energy minimization process, the selected model for the binding site was the one containing a shape-based volume combined with a hydrogen bond acceptor feature, located in the region of the main interaction with the amino acid Ser337, and for the allosteric site a standard docking protocol was developed for non-covalent binding [38]. 

**Figure 4:**
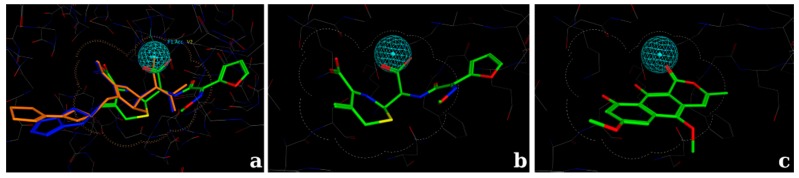
3D shape-based volume surrounding the core interactions of the chemical structures combined with a hydrogen bond acceptor feature (cyan sphere). (**a**) Superposition of three inhibitors in its crystallographic pose: cefuroxime (sticks in green) biapenem (sticks in blue) and tebipenem (sticks in orange); (**b**) Crystallographic pose of the inhibitor cefuroxime evidencing that the core betalactamic ring is comprised by the shape-based volume; (**c**) Docking pose of the isocoumarin paepalantine evidencing that the main chemical structure fits inside the 3D shape-based volume used during docking calculation.

The docking calculations for the active site consisted of the use of a 3D shape-based volume surrounding the core interactions combined with a hydrogen bond acceptor feature (Figure 4). Then, this information was used as a placement method setting using the pharmacophore model as the search engine, and then the conformations of the ligand were filtered to enforce shape occupancy, according to the model that was developed. The placement method used during docking calculations generate poses from ligand conformations. A pose is defined as a specific conformation of the ligand with a specific translational and rotational orientation in the active site [43].

A pharmacophore model contains Boolean expressions to encode concepts representing a hydrogen bond donor, hydrogen bond acceptor, hydrophobic, aromatic, anionic, etc., indicated by colored spheres named pharmacophore features [39,44]. One can also add some restrictions adding exclusion spheres or adding a shape-based model to delimit the search, according to the proposed investigation conducted [38]. For the proposed docking model in the active site, a hydrogen bond acceptor feature was then selected to find appropriate chemical groups to interact with the hydroxyl of the serine amino acid (Figure 4).

#### 2.2.3. Docking Calculations for the Isocoumarin Paepalantine

The docking score values for paepalantine were in a range similar to the reference inhibitor cefuroxime: S = −5.7 to −5.4 for the active site and S = −6.4 to −5.3 for the allosteric site. The values are in accordance with the reference values, S = −7.8 to −6.5 for the active site and S = −6.8 to −5.8 for the allosteric site, evidencing that the proposed model was able to describe the main interactions. For more information about the docking score values, access the available supplementary material. 

## 3. Discussion

Despite many years of experience in fighting *H. pylori*, the ideal model for treatment against this infection is yet to be discovered [2]. Mechanisms of drug resistance, increased side effects, low adherence, and the high cost of antibiotic therapy increase treatment failure. Antibiotics are not the only factor involved in the successful eradication of *H. pylori*. The micro-environment created by this bacterium is a further factor that influences the eradication rate. Specifically, the most severe clinical manifestation associated with certain *H. pylori* strains is most likely due to a higher degree of inflammation caused by these bacteria [45,46,47].

In this context, alternative treatment approaches must be developed and the research of natural products is a valuable resource in drug discovery [9]. The use of substances with both antibiotic and anti-oxidant activity has been suggested to increase treatment efficiency by reducing the inflammation and oxidative stress in the gastric mucosa [48,49]. Coumarins compose a group of natural compounds that are found in several plant sources and show excellent pharmaceutical potential, exhibiting anti-inflammatory, anti-oxidant, antimicrobial, anticoagulant, antitumor, and antiviral activity, amongst others [50]. Previous studies with the isocoumarin paepalantine have shown anti-oxidant [30], anti-inflammatory [32], and antibacterial potential [29] for this substance.

In the present study, we evaluated the anti-*H. pylori* effects of paepalantine, which exhibited significant activity, with a MIC of 128 µg/mL and a MBC of 256 µg/mL. Structure-activity studies with coumarins suggest that the presence of antimicrobial activity is directly related to the lipophilicity and planar structure of the molecule [22,51]. Its lipophilic character is likely responsible for its entrance into bacterial membranes [52]. Additionally, the catechol-like arrangement provided by the 9-OH and 10-OH groups in paepalantine and its planar structure, together with its lipophilic characteristics, are likely responsible for the antibiotic capacity of paepalantine [27,29]. 

Some authors have observed morphological changes in *H. pylori* caused by subinhibitory concentrations of substances with antibiotic activity. The analysis of β-lactam antibiotics via transmission electron microscopy (TEM) has shown a strong link of bacterial penicillin-binding proteins (PBPs), characterized by large spherical cells with few cytoplasmic elements and eventual membrane rupture, thus suggesting PBP63 inhibition as the main mechanism responsible for these changes [2,34]. Other types of changes have also been observed, including the formation of vacuoles, membrane swelling, and cell destruction after treatment with a synthetic molecule leading to the development of a spherical shape by the bacterium is a passive process that does not require protein synthesis and is one of the stages of cell death [4].

The SEM morphological analysis of *H. pylori* subjected to the sub-MIC of paepalantine revealed spherical, irregular shapes, which were related to membrane protein inhibition.

The morphological changes observed in *H. pylori* after exposure to the sub-MIC of paepalantine included swelling of the bacillary forms, bleb formation on the cell surface, and the emergence of spherical shapes. These observations suggest that the target of paepalantine activity may be located at the cell surface, acting as a permeability barrier. The bactericidal mechanism of paepalantine against *H. pylori* might then be found in the disruption of the permeability barrier within cell membranes.

**Figure 5:**
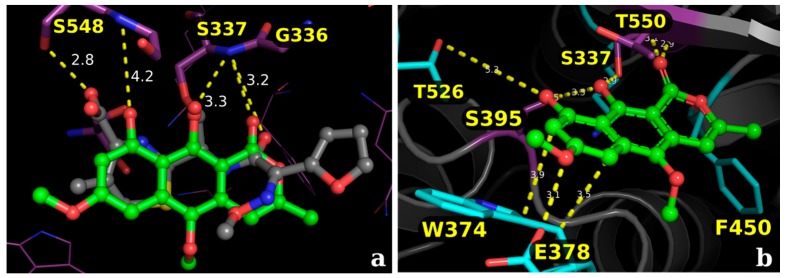
(**a**) Superposition of the crystallographic pose of the inhibitor cefuroxime (sticks in gray) and the binding modes of paepalantine (sticks in green), evidencing the similar placement in the active site; (**b**) Proposed binding mode for the isocoumarin paepalantine in the PBP active site: sticks in purple represents the main interactions and sticks in cyan the secondary interactions.

Based on the results from the experimental tests (Scanning electron microscopy), in silico studies comprised by docking analysis were conducted to investigate the potential interactions of the metabolite paepalantine in the binding site of the penicillin-binding proteins (PBP) [42,53]. The analyzed binding pocket includes the active site and the allosteric site. The active site calculations resulted in a docking pose for paepalantine with similar placement in the binding site to the reference inhibitor (cefuroxime). This docking pose evidences potential interactions with key residues, similar to other carbapenemic inhibitors near the residues Thr550, Ser548, and Ser395. It is also observed that secondary interactions with the amino acids Ala551, Gln452, Tyr561, and Trp374 were accessed during ring stabilization (Figure 5).

**Figure 6:**
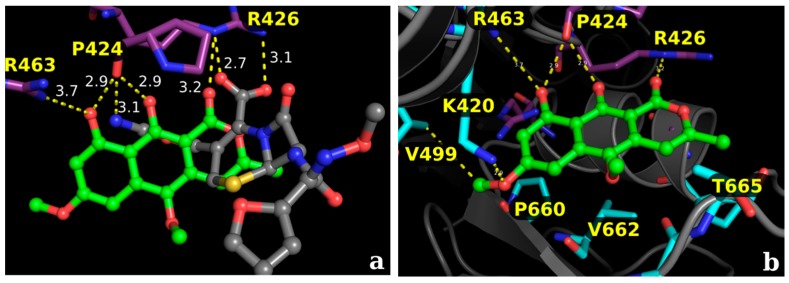
Proposed docking conformations for the isocoumarin paepalantine in the PBP allosteric site: sticks in purple represents the main interactions and sticks in cyan the secondary interactions. (**a**) Superposition of the crystallographic pose of the inhibitor cefuroxime (sticks in gray) and the docking conformation of paepalantine (sticks in green), evidencing that paepalantine can be buried deeply inside the pocket; (**b**) Proposed binding mode for paepalantine in the allosteric site: sticks in purple represents the main interactions and sticks in cyan the secondary interactions.

The analysis of the allosteric site evidences that paepalantine can be buried deep inside its pocket and still accesses the main core interactions of the inhibitor cefuroxime in its non-covalent binding pattern. The hydroxyl groups of paepalantine are near a region of interaction with the key amino acids Pro424 and Arg463. The carbonyl group interacts with the nitrogen atom of Arg426 (Figure 6).

**Figure 7:**
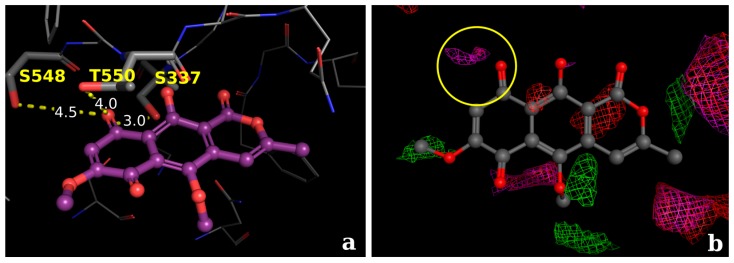
(**a**) Proposed docking conformations for the naphthoquinone derivative (5-Methoxy-3,4-dehydroxanthomegnin) in the PBP active site. (**b**) Molecular interaction field analysis, evidencing the group probes for carboxylate (E = 6.2 kcal·mol^−1^; pink probes) and hydroxyl group (E = 5.8 kcal·mol^−1^; red probes).

**Figure 8:**
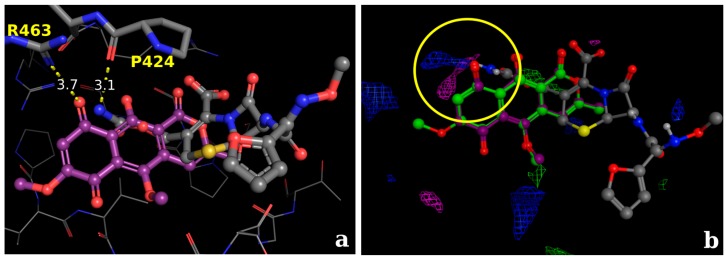
(**a**) Superposition of the naphthoquinone derivative (5-Methoxy-3,4-dehydroxanthomegnin) and the inhibitor cefuroxime, suggesting that both ligands could interact via H-bond with the amino acid, Pro424, with a distance of 3.1 Å in the PBP allosteric site; (**b**) Molecular interaction field analysis, evidencing the group probes for carboxylate (E = 6.2 kcal·mol^−1^; pink probes) and the amino group of cefuroxime (E = 5.4 kcal·mol^−1^; blue probes), highlighting the potential of interaction in both situations.

Comparing the antibacterial activity of the metabolite paepalantine with other active compounds and analogues (e.g., 5-methoxy-3,4-dehydroxanthomegnin, Figure 7 and Figure 8), which has a 2-fold higher antibacterial property against *H. pylori* [54]), the docking analyses corroborates that the naphthoquinone derivative (5-Methoxy-3,4-dehydroxanthomegnin) (Figure 7a) is better stabilized in the binding site than paepalantine. This inference is based on the observation that the carboxyl group of the naphthoquinone ring is a stronger hydrogen-bond acceptor than the corresponding hydroxyl group on paepalantine. In this process, two hydrogen bonds take place between the naphthoquinone ring and the amino acid residues, Thr550 and Ser337 (distances of 4 and 3 Å, respectively). Based on the analyses of the interaction potentials at the active site (yellow circle, Figure 7b, it is proposed that the establishment of an H-bond with the carboxyl group (purple electrostatic grid) is more plausible than the corresponding interaction with the hydroxyl group (small red electrostatic grid). 

Analyses concerning the allosteric site suggests that the interaction of the naphthoquinone derivative (5-Methoxy-3,4-dehydroxanthomegnin) (Figure 8a) with the amino acid Arg463 via an H-bond (distance of 3.7 Å). Although one may consider the ionic interaction between the carboxylate group and the arginine residue as reinforced by an additional H-bond, the considerable distance between these groups would reflect a very weak interaction. A comparison between the inhibitors cefuroxime and paepalantine at the same site suggests that both ligands could interact via H-bond with the amino acid, Pro424, with a distance of 3.1 Å (Figure 8a). Analyses of the electrostatic grid (Figure 8b, yellow circle) suggest that both interactions are favorable at this region, since appropriate distances between the interacting groups are observed.

## 4. Materials and Methods

### 4.1. Plant Material

Paepalanthus bromelioides was collected at Serra do Cipo, Minas Gerais State, Brazil, and identified by Dr. Paulo Takeo Sano from the Institute of Biosciences, University of Sao Paulo. The voucher specimen (CFSC, 13839) is on file at the herbarium in the Department of Botany, Institute of Biosciences, University of Sao Paulo, Brazil. 

### 4.2. Chemicals

Dimethyl sulfoxide (DMSO), peroxidase (77332), and deoxyribose (121649) were purchased from Sigma Chemical Co. (St. Louis, MO, USA). Propylene glycol was purchased from Neon Comercial Ltda. (São Paulo, SP, Brazil). Paepalantine was obtained according to the procedure previously reported by Vilegas et al. (1990) [27], and the stock solution was prepared at 10 mg/mL in propylene glycol [55]. The highest absorption of paepalantine was at 265 nm.

### 4.3. In Vitro Studies

#### 4.3.1. Evaluation of Anti-*H. pylori* Activity

Anti-*H. pylori* activity was assessed by determining the minimum inhibitory concentration (MIC) using the spectrophotometric method of broth micro dilution according to the guidelines of the Clinical and Laboratory Standards Institute (CLSI) (M7-A6, 2003). Microplate wells were filled with 100 µL of liquid growth medium (brain-heart infusion supplemented with 10% fetal calf serum) containing different concentrations of test agent (16–1024 µg/mL). Another 100 µL containing *H. pylori* (ATCC 43504) suspension (approximately 10^6^–10^7^ bacteria per mL) was added to each well. Absorbance was read in a spectrophotometer at a wavelength of 620 nm, and the microplates were then incubated at 36–37°C in the presence of 10% CO_2_ for 72 h. After incubation, the plate well contents were homogenized, and a new spectrophotometric reading was performed under the abovementioned conditions. The tests were performed in triplicate and repeated at least three times, together with growth controls (absence of sample) and negative controls (culture medium containing different concentrations of test agent to control for color). The MIC was determined graphically and was defined as the lowest concentration of antibacterial agent at which there was a sharp decline (90%) in absorbance. Amoxicillin and metronidazole were used as standards.

#### 4.3.2. Morphological Analysis of *H. pylori* Bacterial Cells

A morphological analysis of *H. pylori* bacteria after exposure to the MIC and a sub-MIC was performed by scanning electron microscopy (SEM). For sample preparation, culture medium containing bacteria exposed to paepalantine was aliquoted and centrifuged at 4000 rpm for 5 min. The supernatant was discarded, and 1 mL of 0.1 M sodium cacodylate buffer (pH 7.2) was added. After centrifugation, the cell pellet was resuspended with 200 µL of 0.1 M sodium cacodylate buffer (pH 7.2). One aliquot was placed in the center of a cover slip and allowed to dry. The material was then fixed with 2.5% glutaraldehyde in 0.1 M sodium cacodylate buffer (pH 7.2) for 30 min. Finally, the sample was dehydrated with alcohol, and the material was sputter coated for analysis in a JEOL^®^ JSM-6610LV scanning electron microscope (Tokyo, Japan) at an accelerating voltage of 10 kV [56,57,58].

### 4.4. Computational Methods

Molecular docking calculations were performed using the Molecular Operating Environment suite (MOE) [39].The protein structure was loaded into MOE software using the "Load PDB File" panel. The water chains were analyzed regarding their relevance for docking, and then were further deleted since no relevant contribution was observed. Structural issues with protein were corrected using the “Structure Preparation” panel, adding hydrogens and correcting structural issues in the protein. Partial charges and hydrogen bond optimization were conducted with the force field “MMFF94x” and the “Protonate 3D” panel, respectively. Two docking models were developed: one for the active site and another for the allosteric site.

The docking model for the active site was developed to investigate the potential of binding for small inhibitors, such as the investigated metabolite, and thus an appropriate protein-ligand complex with a small ligand inside was selected. Since the active site comprises an acylation mechanism, this chemical bond was undone, restoring the original hydroxyl group of the serine amino acid (Ser337) and applying the described methods for protein preparation and hydrogen bond optimization [39]. The original cavity of the active site was then preserved and optimized for small inhibitors since it was previously complexed with a small inhibitor, cefuroxime. Then, a volume shape-based using a pharmacophore constraint was established to delimit the main binding site interactions. The pharmacophore search engine was set as the placement method during docking. 

For the allosteric site, the placement method was set to “triangle matcher”. The selected scoring function was set to “London dG” and the free energy of binding was estimated using the force field-based scoring function “GBVI/WSA” for both models. The three-dimensional structures of the penicillin-binding proteins were obtained from the RCSB Protein Data Bank [36]. The chemical structures were drawn with Marvin Sketch software [59], the partial charges calculated with MOE [39], minimized with Balloon software [60] and the conformer generation calculated with OMEGA software [61,62].

### 4.5. Statistical Analysis

Data are expressed as the mean ± standard deviation and were subjected to analysis of variance (ANOVA) and linear regression. The significance level was set at *p* < 0.05.

## 5. Conclusions

Beta-lactam antibiotics are a class of broad-spectrum antibiotics introduced in the later stages of World War II and represents one of the most important contributions to medical science in recent history. The beta-lactams remain the most widely utilized antibiotics for several reasons, such as high effectiveness, low costs, ease of delivery, and minimal side effects [63]. One of the most common bacterial resistance mechanism is the production of enzymes that degrade or modify the antibiotic before it can reach the protein’s *binding* site. The investigation of natural products as a source of new scaffolds for drug development is an interesting strategy and must be investigated.

The data from the present study indicates that paepalantine exhibits significant anti-*H. pylori* activity, most likely by inhibiting membrane protein synthesis. Thus, paepalantine may be a promising molecule for the treatment and prevention of diseases caused by *H. pylori*.

The initial molecular modeling docking calculations suggests that the isocoumarin paepalantine should be investigated for PBP inhibition, since one of the main mechanisms that could help its effectiveness against the PBPs, based on the results of the electron microscopy, is that it will not contain the beta-lactamic ring, and thus the beta-lactamases will be ineffective [34,63,64]. Identification of novel scaffolds for potential anti-*H. pylori* agents based on molecular modeling techniques are important strategies to implement during the drug design process since the actual multi-therapy with amoxicillin and clarithromycin associated with a proton pump inhibitor such as omeprazole is no longer effective due to the prevalence of antibiotic resistance [65,66].

However, other mechanisms of action cannot be ruled out, such as the interruption of *H. pylori* colonization by the inactivation of bacterial DNA gyrase, which is a common mechanism among coumarin derivatives and urease inhibition.

## Figures and Tables

**Table 1:** Available PBP2x protein complexes from The Protein Data Bank.

PDB Code	Target	Inhibitor	Class	Reference
1QMF	PBP2x	Cefuroxime	Cephalosporin	doi:10.1006/jmbi.2000.3740
2ZC4	PBP2x	Tebipenem	Carbapenem	doi:10.1128/AAC.01456-07
2ZC3	PBP2x	Biapenem	Carbapenem	doi:10.1128/AAC.01456-07

## Supplementary Materials

Supplementary materials are available online.

## References

[1] Ladeira M.S.P., Salvadori D.M.F., Rodrigues M.A.M. (2003). Biopatologia do *Helicobacter pylori*. J. Bras. Patol. Med. Lab..

[2] Kusters J.G., van Vliet A.H.M., Kuipers E.J. (2006). Pathogenesis of *Helicobacter pylori* Infection. Clin. Microbiol. Rev..

[3] Malaty H.M. (2007). Epidemiology of *Helicobacter pylori* infection. Best Pract. Res. Clin. Gastroenterol..

[4] Dai G., Cheng N., Dong L., Muramatsu M., Xiao S., Wang M.W., Zhu D.X. (2005). Bactericidal and morphological effects of NE-2001, a novel synthetic agent directed against *Helicobacter pylori*. Antimicrob. Agents Chemother..

[5] Wang G., Alamuri P., Maier R.J. (2006). The diverse antioxidant systems of *Helicobacter pylori*. Mol. Microbiol..

[6] Allen L.A.H., Beecher B.R., Lynch J.T., Rohner O.V., Wittine L.M. (2005). *Helicobacter pylori* disrupts NADPH oxidase targeting in human neutrophils to induce extracellular superoxide release. J. Immunol..

[7] Wu Y., Antony S., Meitzler J.L., Doroshow J.H. (2014). Molecular mechanisms underlying chronic inflammation-associated cancers. Cancer Lett..

[8] Eusebi L.H., Zagari R.M., Bazzoli F. (2014). Epidemiology of *Helicobacter pylori* Infection. Helicobacter.

[9] Miftahussurur M., Yamaoka Y. (2015). Appropriate first-line regimens to combat *Helicobacter pylori* antibiotic resistance: An asian perspective. Molecules.

[10] Nishizawa T., Suzuki H., Suzuki M., Takahashi M., Hibi T. (2012). Proton pump inhibitor-amoxicillin-clarithromycin versus proton pump inhibitor-amoxicillin-metronidazole as first-line *Helicobacter pylori* eradication therapy. J. Clin. Biochem. Nutr..

[11] Jadhav S.G., Meshram R.J., Gond D.S., Gacche R.N. (2013). Inhibition of growth of *Helicobacter pylori* and its urease by coumarin derivatives: Molecular docking analysis. J. Pharm. Res..

[12] Bonacorsi C., Raddi M.S.G., Carlos I.Z., Sannomiya M., Vilegas W. (2009). Anti-*Helicobacter pylori* activity and immunostimulatory effect of extracts from Byrsonima crassa Nied. (Malpighiaceae). BMC Complement. Altern. Med..

[13] Ecclissato C., Marchioretto M.A.M., Mendonca S., Godoy A.P.O., Guersoni R.A., Deguer M., Piovesan H., Ferraz J.G.P., Pedrazzoli J. (2002). Increased Primary Resistance to Recommended Antibiotics Negatively Affects *Helicobacter pylori* Eradication. Helicobacter.

[14] Prasertpetmanee S., Mahachai V., Vilaichone R.K. (2013). Improved efficacy of proton pump inhibitor-amoxicillin-clarithromycin triple therapy for *Helicobacter pylori* eradication in low clarithromycin resistance areas or for tailored therapy. Helicobacter.

[15] Abu-Qatouseh L., Abu-Sini M., Mayyas A., Al-Hiari Y., Darwish R., Aburjai T. (2017). Synthesis of New Nitrofluoroquinolone Derivatives with Novel Anti-Microbial Properties against Metronidazole Resistant *H. pylori*. Molecules.

[16] Amin M., Anwar F., Naz F., Mehmood T., Saari N. (2013). Anti-*Helicobacter pylori* and urease inhibition activities of some traditional medicinal plants. Molecules.

[17] Patridge E., Gareiss P., Kinch M.S., Hoyer D. (2016). An analysis of FDA-approved drugs: Natural products and their derivatives. Drug Discov. Today.

[18] Davoine F., Lacy P. (2014). Eosinophil cytokines, chemokines, and growth factors: Emerging roles in immunity. Front. Immunol..

[19] Rob J.A., Tollefsen S., Helgeland L. (1997). A Rapid and Highly Sensitive Chromogenic Microplate Assay for Quantification of Rat and Human Prothrombin. Anal. Biochem..

[20] Grover J., Jachak S.M. (2015). Coumarins as privileged scaffold for anti-inflammatory drug development. RSC Adv..

[21] Paya M., Goodwin P.A., De Las Heras B., Hoult J.R.S. (1994). Superoxide scavenging activity in leukocytes and absence of cellular toxicity of a series of coumarins. Biochem. Pharmacol..

[22] Kayser O., Kolodziej H. (1999). Antibacterial activity of simple coumarins: Structural requirements for biological activity. Z. Naturforsch. C.

[23] Ribeiro C.V.C., Kaplan M.A.C. (2002). Tendências evolutivas de famílias produtoras de cumarinas em angiospermae. Quim. Nova.

[24] Hardy C.D., Cozzarelli N.R. (2003). Alteration of Escherichia coli Topoisomerase IV to Novobiocin Resistance. Antimicrob. Agents Chemother..

[25] Lee J.H., Kim Y.G., Cho H.S., Ryu S.Y., Cho M.H., Lee J. (2014). Coumarins reduce biofilm formation and the virulence of Escherichia coli O157:H7. Phytomedicine.

[26] Lin H.C., Tsai S.H., Chen C.S., Chang Y.C., Lee C.M., Lai Z.Y., Lin C.M. (2008). Structure–activity relationship of coumarin derivatives on xanthine oxidase-inhibiting and free radical-scavenging activities. Biochem. Pharmacol..

[27] Vilegas W., Roque N.F., Salatino A., Giesbrecht A.M., Davino S. (1990). Isocoumarin from Paepalanthus bromelioides. Phytochemistry.

[28] Devienne K.F., Raddi M.S.G. (2002). Screening for antimicrobial activity of natural products using a microplate photometer. Braz. J. Microbiol..

[29] Ferrazzoli Devienne K., Gonçalves Raddi M.S., Gomes Coelho R., Vilegas W. (2005). Structure-antimicrobial activity of some natural isocoumarins and their analogues. Phytomedicine.

[30] Kitagawa R.R., Raddi M.S.G., Khalil N.M., Vilegas W., da Fonseca L.M. (2003). Effect of the isocoumarin paepalantine on the luminol and lucigenin amplified chemiluminescence of rat neutrophils. Biol. Pharm. Bull..

[31] Devienne K.F., Cálgaro-Helena A.F., Dorta D.J., Prado I.M.R., Raddi M.S.G., Vilegas W., Uyemura S.A., Santos A.C., Curti C. (2007). Antioxidant activity of isocoumarins isolated from Paepalanthus bromelioides on mitochondria. Phytochemistry.

[32] Di Stasi L.C., Camuesco D., Nieto A., Vilegas W., Zarzuelo A., Galvez J. (2004). Intestinal anti-inflammatory activity of paepalantine, an isocoumarin isolated from the capitula of Paepalanthus bromelioides, in the trinitrobenzenesulphonic acid model of rat colitis. Planta Med..

[33] Varanda E.A., Devienne K.F., Raddi M.S.G., Furuya E.M., Vilegas W. (2004). Mutagenicity of paepalantine dimer and glycoside derivatives from Paepalanthus bromelioides. Toxicol. In Vitro.

[34] DeLoney C.R., Schiller N.L. (1999). Competition of various beta-lactam antibiotics for the major penicillin-binding proteins of *Helicobacter pylori*: Antibacterial activity and effects on bacterial morphology. Antimicrob. Agents Chemother..

[35] Berry V., Jennings K., Woodnutt G. (1995). Bactericidal and Morphological Effects of Amoxicillin on *Helicobacter-pylori*. Antimicrob. Agents Chemother..

[36] Bernstein F.C., Koetzle T.F., Williams G.J., Meyer E.F., Brice M.D., Rodgers J.R., Kennard O., Shimanouchi T., Tasumi M. (1977). The Protein Data Bank. A computer-based archival file for macromolecular structures. Eur. J. Biochem..

[37] Kirchmair J., Markt P., Distinto S., Schuster D., Spitzer G.M., Liedl K.R., Langer T., Wolber G. (2008). The Protein Data Bank (PDB), Its Related Services and Software Tools as Key Components for In Silico Guided Drug Discovery. J. Med. Chem..

[38] Mahasenan K.V., Molina R., Bouley R., Batuecas M.T., Fisher J.F., Hermoso J.A., Chang M., Mobashery S. (2017). Conformational Dynamics in Penicillin-Binding Protein 2a of Methicillin-Resistant *Staphylococcus aureus*, Allosteric Communication Network and Enablement of Catalysis. J. Am. Chem. Soc..

[39] Chemical Computing Group Inc. (2013). Molecular Operating Environment (MOE) User Manual.

[40] Vilar S., Cozza G., Moro S. (2008). Medicinal chemistry and the molecular operating environment (MOE): Application of QSAR and molecular docking to drug discovery. Curr. Top. Med. Chem..

[41] Kitchen D.B., Decornez H., Furr J.R., Bajorath J. (2004). Docking and scoring in virtual screening for drug discovery: Methods and applications. Nat. Rev. Drug Discov..

[42] Plewczynski D., Łaźniewski M., Augustyniak R., Ginalski K. (2011). Can we trust docking results? Evaluation of seven commonly used programs on PDBbind database. J. Comput. Chem..

[43] Selzer P.M. (2009). Antiparasitic and Antibacterial Drug Discovery: From Molecular Targets to Drug Candidates.

[44] Vuorinen A., Schuster D. (2015). Methods for generating and applying pharmacophore models as virtual screening filters and for bioactivity profiling. Methods.

[45] Ayala G., Galván-Portillo M., Chihu L., Fierros G., Sánchez A., Carrillo B., Román A., López-Carrillo L., Silva-Sánchez J., Study Group (2011). Resistance to Antibiotics and Characterization of *Helicobacter pylori* Strains Isolated from Antrum and Body from Adults in Mexico. Microb. Drug Resist..

[46] Graham D.Y., Shiotani A. (2008). New concepts of resistance in the treatment of *Helicobacter pylori* infections. Nat. Clin. Pract. Gastroenterol. Hepatol..

[47] Azab A., Nassar A., Azab A.N. (2016). Anti-Inflammatory Activity of Natural Products. Molecules.

[48] Vale F.F. (2014). Overview of the phytomedicine approaches against *Helicobacter pylori*. World J. Gastroenterol..

[49] Vítor J.M.B., Vale F.F. (2011). Alternative therapies for *Helicobacter pylori*: Probiotics and phytomedicine. FEMS Immunol. Med. Microbiol..

[50] Kostova I., Bhatia S., Grigorov P., Balkansky S., Parmar V.S., Prasad A.K., Saso L. (2011). Coumarins as Antioxidants. Curr. Med. Chem..

[51] Yang L., Ding W., Xu Y., Wu D., Li S., Chen J., Guo B. (2016). New Insights into the Antibacterial Activity of Hydroxycoumarins against Ralstonia solanacearum. Molecules.

[52] Rauckman B.S., Tidwell M.Y., Johnson J.V., Roth B. (1989). 2,4-Diamino-5-benzylpyrimidines and analogues as antibacterial agents. 10. 2,4-Diamino-5-(6-quinolylmethyl)- and -[(tetrahydro-6-quinolyl)methyl]pyrimidine derivatives. Further specificity studies. J. Med. Chem..

[53] Rodrigues R., Mantoani S.P., de Almeida J.R., Pinsetta F.R., Semighini E.P., da Silva V.B., da Silva C.H.T.P. (2012). Estratégias de Triagem Virtual no Planejamento de Fármacos. Rev. Virtual Quím..

[54] Kitagawa R.R., Bonacorsi C., da Fonseca L.M., Vilegas W., Raddi M.S.G. (2012). Anti-*Helicobacter pylori* activity and oxidative burst inhibition by the naphthoquinone 5-methoxy-3,4-dehydroxanthomegnin from *Paepalanthus latipes*. Rev. Bras. Farmacogn..

[55] Damasceno J.P.L., dos Giuberti C.S., de Gonçalves R.C.R., Kitagawa R.R. (2015). Preformulation study and influence of DMSO and propylene glycol on the antioxidant action of isocoumarin paepalantine isolated from Paepalanthus bromelioides. Rev. Bras. Farmacogn..

[56] Chakraborti S., Bhattacharya S., Chowdhury R., Chakrabarti P. (2013). The molecular basis of inactivation of metronidazole-resistant *Helicobacter pylori* using polyethyleneimine functionalized zinc oxide nanoparticles. PLoS ONE.

[57] Fischer C.L., Walters K.S., Drake D.R., Blanchette D.R., Dawson D.V., Brogden K.A., Wertz P.W. (2013). Sphingoid Bases Are Taken Up by *Escherichia coli* and *Staphylococcus aureus* and Induce Ultrastructural Damage. Skin Pharmacol. Physiol..

[58] Claverie-Martin F., Diaz-Torres M.R., Geoghegan M.J. (1988). Chemical composition and ultrastructure of wild-type and white mutantAspergillus nidulans conidial walls. Curr. Microbiol..

[59] ChemAxon Marvin Sketch. https://www.chemaxon.com/products/marvin/marvinsketch/.

[60] Vainio M.J., Johnson M.S. (2007). Generating conformer ensembles using a multiobjective genetic algorithm. J. Chem. Inf. Model..

[61] Hawkins P.C., Skillman A.G., Warren G.L., Ellingson B.A., Stahl M.T. (2010). Conformer generation with OMEGA: Algorithm and validation using high quality structures from the Protein Databank and Cambridge Structural Database. J. Chem. Inf. Model..

[62] OMEGA 2.5.1.4: OpenEye Scientific Software, Santa Fe, NM. http://www.eyesopen.com.

[63] Wilke M.S., Lovering A.L., Strynadka N.C. (2005). β-Lactam antibiotic resistance: A current structural perspective. Curr. Opin. Microbiol..

[64] Kumar K.M., Anbarasu A., Ramaiah S. (2014). Molecular docking and molecular dynamics studies on β-lactamases and penicillin binding proteins. Mol. Biosyst..

[65] Cho S., Im H., Lee K.Y., Chen J., Kang H.J., Yoon H.J., Min K.H., Lee K.R., Park H.J., Lee B.J. (2016). Identification of novel scaffolds for potential anti-*Helicobacter pylori* agents based on the crystal structure of *H. pylori* 3-deoxy-d-manno-octulosonate 8-phosphate synthase (HpKDO8PS). Eur. J. Med. Chem..

[66] Fock K.M., Graham D.Y., Malfertheiner P. (2013). *Helicobacter pylori* research: Historical insights and future directions. Nat. Rev. Gastroenterol. Hepatol..

