# Supplementary material for: Anti-Helicobacter pylori Activity of Isocoumarin Paepalantine: Morphological and Molecular Docking Analysis
Source: Molecules. 2017 May 12;22(5):786. doi: 10.3390/molecules22050786 (PMC6154667; doi:10.3390/molecules22050786)
Supplement: Supplementary file 1 [file molecules-22-00786-s001.pdf]

Supplementary Material

Anti-*Helicobacter pylori* Activity of Isocoumarin Paepalantine: Morfhological and Molecular Docking Analysis

João Paulo Damasceno, Ricardo Rodrigues, Rita Gonçalves and Rodrigo Kitagawa

1. Supplementary Material

1.1. Chemical Structures Mentioned in the Article.

Chemical Structures		
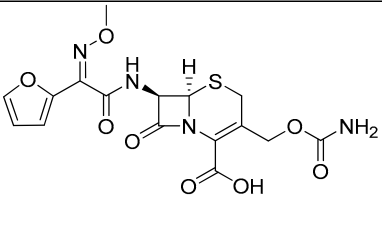 (Cefuroxime)	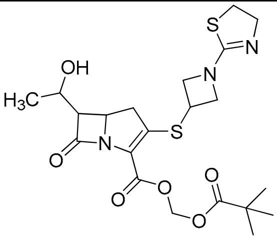 (Tebipenem)	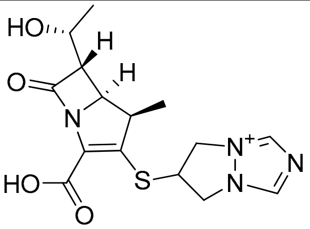 (Biapenem)
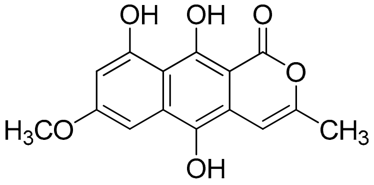 (9,10-dihydroxy-5,7-dimethoxy-3-methyl-1H-naphtho[2,3-c]pyran-1-one)	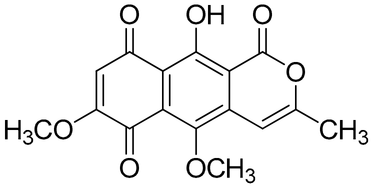 (5-methoxy-3,4-dehydroxanthomegnin)	

1.2. Docking Validation

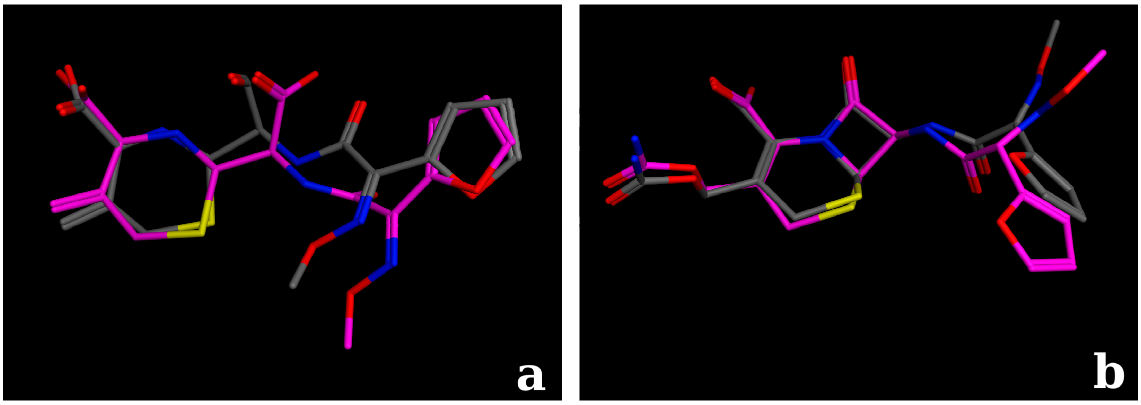

Figure S1: Redocking poses of the inhibitor cefuroxime. (A), Redocking of the inhibitor in the PBP binding site (RMSD = 1.14 Å). (B), Redocking of the inhibitor in the PBP allosteric site (RMSD = 1.31 Å). The carbon atoms with sticks in pink represents the docking pose and the carbon atoms with sticks in gray represents the crystallographic pose.

1.3. Docking Calculations for the Active Site

Table S1. Score values for the top 10 docking poses for the inhibitor cefuroxime in the active site during the redocking process.

Pose	Score (S)*	RMSD**	RMSD refine¤	E conf§	E placeø	E score1Φ	E refineϕ	E score2ω
1	-7.818	1.141	1.874	-33.038	-77.115	-15.144	-21.643	-7.818
2	-7.041	3.656	1.431	-44.349	-115.528	-11.171	-19.530	-7.041
3	-6.546	3.850	1.179	-39.092	-106.030	-11.340	-15.598	-6.546
4	-6.546	3.850	1.196	-39.094	-101.360	-11.447	-15.597	-6.546
5	-6.532	3.709	1.309	-39.491	-91.749	-13.250	-15.857	-6.532
6	-6.525	3.708	1.597	-39.489	-85.026	-11.215	-15.856	-6.525
7	-6.517	3.710	1.589	-39.468	-92.130	-11.331	-15.871	-6.517
8	-6.517	3.710	1.622	-39.472	-84.370	-12.420	-15.873	-6.517
9	-6.516	3.710	1.619	-39.484	-84.709	-12.568	-15.866	-6.516
10	-6.509	3.744	2.142	-35.205	-82.400	-11.529	-16.423	-6.509

* - the final score; ** - the root mean square deviation of the pose, in Å, from the original ligand. ¤ - the root mean square deviation between the pose before refinement and the pose after refinement; § - the energy of the conformer; ø - the score from the placement stage; Φ,ω - represents the score from rescoring stages 1 and 2; ϕ - describes the score from the refinement stage (sum of the van der Waals electrostatics and solvation energies).

Table S2. Score values for the top 10 docking poses for the isocoumarin peapalantine in the active site.

Pose	Score (S)*	RMSD refine¤	E conf§	E placeø	E score1Φ	E refineϕ	E score2ω
1	-5.705	1.201	75.296	-68.997	-11.402	-15.490	-5.705
2	-5.705	2.127	75.301	-55.444	-9.214	-15.485	-5.705
3	-5.693	1.094	75.299	-61.414	-10.341	-15.486	-5.693
4	-5.690	1.415	71.336	-65.669	-8.922	-17.828	-5.690
5	-5.679	1.336	71.316	-79.898	-9.9156	-17.807	-5.679
6	-5.597	1.652	74.992	-39.537	-9.402	-15.174	-5.597
7	-5.596	1.854	74.987	-40.647	-9.940	-15.164	-5.596
8	-5.591	1.556	74.994	-57.335	-12.297	-15.180	-5.591
9	-5.485	2.783	77.867	-66.109	-10.667	-17.288	-5.485
10	-5.480	3.102	77.888	-15.893	-10.381	-17.317	-5.480

* - the final score; ¤ - the root mean square deviation between the pose before refinement and the pose after refinement; § - the energy of the conformer; ø - the score from the placement stage; Φ,ω - represents the score from rescoring stages 1 and 2; ϕ - describes the score from the refinement stage (sum of the van der Waals electrostatics and solvation energies).

Table S3. Score values for the top 10 docking poses for the metabolite 5-Methoxy-3,4-dehydroxanthomegnin in the active site.

Pose	Score (S)*	RMSD refine¤	E conf§	E placeø	E score1Φ	E refineϕ	E score2ω
1	-6.305	1.525	-16.980	-80.109	-18.793	-22.164	-6.305
2	-6.184	1.377	-14.998	-86.696	-19.142	-21.271	-6.184
3	-6.109	1.273	-15.804	-77.075	-18.420	-23.604	-6.109
4	-6.023	1.362	-17.601	-79.428	-18.572	-28.933	-6.023
5	-5.911	1.124	-11.696	-100.842	-17.871	-26.856	-5.911
6	-5.862	2.373	-15.216	-81.480	-19.006	-28.927	-5.862
7	-5.835	1.331	-16.954	-93.482	-18.283	-30.607	-5.835
8	-5.805	2.351	-14.908	-78.041	-18.913	-26.915	-5.805
9	-5.787	1.371	-14.832	-81.519	-19.002	-28.085	-5.787
10	-5.604	1.115	-15.304	-77.159	-17.861	-28.272	-5.604

* - the final score; ¤ - the root mean square deviation between the pose before refinement and the pose after refinement; § - the energy of the conformer; ø - the score from the placement stage; Φ,ω - represents the score from rescoring stages 1 and 2; ϕ - describes the score from the refinement stage (sum of the van der Waals electrostatics and solvation energies).

1.4. Docking Calculations for the Allosteric Site

Table S4. Score values for the top 10 docking poses for the inhibitor cefuroxime in the allosteric site during the redocking process.

Pose	Score (S)*	RMSD**	RMSD refine¤	E conf§	E placeø	E score1Φ	E refineϕ	E score2ω
1	-6.806	1.311	1.197	1.087	-79.843	-2.441	-39.652	-6.806
2	-6.998	1.347	1.093	-1.845	-106.419	-2.523	-43.518	-6.998
3	-6.401	2.478	1.261	0.337	-60.514	-2.614	-35.230	-6.401
4	-6.106	2.498	2.780	4.641	-52.746	-2.146	-31.524	-6.106
5	-6.894	2.590	1.119	-3.818	-95.041	-2.385	-43.357	-6.894
6	-6.723	2.731	4.004	5.697	-54.195	-2.284	-40.133	-6.723
7	-6.267	2.963	2.576	-0.284	-63.240	-2.139	-36.171	-6.267
8	-5.243	3.558	1.687	3.304	-59.642	-1.985	-27.236	-5.243
9	-4.812	3.791	1.321	-2.135	-53.562	-1.990	-22.839	-4.812
10	-5.845	3.895	2.077	2.668	-50.305	-2.025	-32.996	-5.845

* - the final score; ** - the root mean square deviation of the pose, in Å, from the original ligand. ¤ - the root mean square deviation between the pose before refinement and the pose after refinement; § - the energy of the conformer; ø - the score from the placement stage; Φ,ω - represents the score from rescoring stages 1 and 2; ϕ - describes the score from the refinement stage (sum of the van der Waals electrostatics and solvation energies).

Table S5. Score values for the top 10 docking poses for the isocoumarin peapalantine in the allosteric site.

Pose	Score (S)*	RMSD refine¤	E conf§	E placeø	E score1Φ	E refineϕ	E score2ω
1	-6.436	2.033	1.967	-64.664	-11.776	-32.946	-6.436
2	-6.177	1.045	2.408	-59.688	-11.796	-31.047	-6.177
3	-5.781	1.572	8.173	-61.789	-12.516	-25.147	-5.781
4	-5.690	1.487	1.233	-56.822	-11.617	-25.023	-5.690
5	-5.624	1.425	1.644	-54.068	-11.374	-24.493	-5.624
6	-5.612	1.753	1.613	-59.797	-12.849	-24.454	-5.612
7	-5.567	0.866	2.210	-75.219	-11.900	-22.564	-5.567
8	-5.435	0.522	6.157	-65.652	-12.002	-22.325	-5.435
9	-5.431	0.612	5.844	-56.090	-11.446	-20.988	-5.431
10	-5.368	1.519	6.703	-53.809	-11.457	-19.581	-5.368

* - the final score; ¤ - the root mean square deviation between the pose before refinement and the pose after refinement; § - the energy of the conformer; ø - the score from the placement stage; Φ,ω - represents the score from rescoring stages 1 and 2; ϕ - describes the score from the refinement stage (sum of the van der Waals electrostatics and solvation energies).

Table S6. Score values for the top 10 docking poses for the metabolite 5-Methoxy-3,4-dehydroxanthomegnin in the allosteric site.

Pose	Score (S)*	RMSD refine¤	E conf§	E placeø	E score1Φ	E refineϕ	E score2ω
1	-6.589	1.850	-15.997	-60.105	-15.857	-33.342	-6.589
2	-6.500	1.235	-16.301	-74.736	-13.925	-32.476	-6.500
3	-6.483	0.937	-16.280	-58.687	-14.254	-32.624	-6.483
4	-6.423	1.921	-15.018	-58.264	-14.869	-26.055	-6.423
5	-6.389	0.772	-14.206	-65.702	-12.693	-25.817	-6.389
6	-6.376	1.197	-13.123	-61.155	-13.432	-25.856	-6.376
7	-6.370	1.376	-13.693	-77.209	-15.358	-31.154	-6.370
8	-6.313	1.099	-15.112	-60.828	-12.537	-22.712	-6.313
9	-6.313	0.772	-14.685	-72.747	-12.720	-26.335	-6.313
10	-6.288	0.986	-16.217	-69.496	-13.998	-31.579	-6.288

* - the final score; ¤ - the root mean square deviation between the pose before refinement and the pose after refinement; § - the energy of the conformer; ø - the score from the placement stage; Φ,ω - represents the score from rescoring stages 1 and 2; ϕ - describes the score from the refinement stage (sum of the van der Waals electrostatics and solvation energies).
